# Insight into cross-talk between intra-amoebal pathogens

**DOI:** 10.1186/1471-2164-12-542

**Published:** 2011-11-02

**Authors:** Gregory Gimenez, Claire Bertelli, Claire Moliner, Catherine Robert, Didier Raoult, Pierre-Edouard Fournier, Gilbert Greub

**Affiliations:** 1 Unité des rickettsies, Faculté de Médecine, Université de la Méditerranée, Marseille, France URMITE CNRS-IRD UMR 6236, Faculté de Médecine, 27 boulevard Jean Moulin, 13385 Marseille Cedex 05, France; 2 Center for Research on Intracellular Bacteria, Institute of Microbiology, University of Lausanne and University Hospital, Bugnon 48, 1011 Lausanne, Switzerland

## Abstract

**Background:**

Amoebae are phagocytic protists where genetic exchanges might take place between amoeba-resistant bacteria. These amoebal pathogens are able to escape the phagocytic behaviour of their host. They belong to different bacterial phyla and often show a larger genome size than human-infecting pathogens. This characteristic is proposed to be the result of frequent gene exchanges with other bacteria that share a sympatric lifestyle and contrasts with the genome reduction observed among strict human pathogens.

**Results:**

We sequenced the genome of a new amoebal pathogen, *Legionella drancourtii*, and compared its gene content to that of a *Chlamydia-*related bacterium, *Parachlamydia acanthamoebae*. Phylogenetic reconstructions identified seven potential horizontal gene transfers (HGTs) between the two amoeba-resistant bacteria, including a complete operon of four genes that encodes an ABC-type transporter. These comparisons pinpointed potential cases of gene exchange between *P. acanthamoebae *and *Legionella pneumophila*, as well as gene exchanges between other members of the *Legionellales *and *Chlamydiales *orders. Moreover, nine cases represent possible HGTs between representatives from the *Legionellales *or *Chlamydiales *and members of the *Rickettsiales *order.

**Conclusions:**

This study identifies numerous gene exchanges between intracellular *Legionellales *and *Chlamydiales *bacteria, which could preferentially occur within common inclusions in their amoebal hosts. Therefore it contributes to improve our knowledge on the intra-amoebal gene properties associated to their specific lifestyle.

## Background

Free-living amoebae are environmental protozoa, which are important predators that contribute to the control of microbial communities [1]. They feed by phagocytosing bacteria and other large, energy-rich particles that are present in their environment [2,3]. Several microorganisms have adapted to become resistant to amoebal phagocytosis and are able to escape the phagocytic pathway and multiply within phagocytic vacuoles [4]. Certain bacteria that live naturally in amoebae have been proposed to be amoebal pathogens [5,6]. These organisms are phylogenetically diverse and belong to *Chlamydiae; *α, β and γ-*proteobacteria; *and *Bacteroidetes *[7,8]. The similarities in the strategies used by phylogenetically distantly related intracellular pathogens are most easily explained by convergent evolution [9]. Recently, Schmitz-Esser *et al*. [10] reported 59 functional domains that are significantly enriched in amoeba-associated bacteria; half of these domains are more frequently associated with eukaryotic proteins. However, the genomic characteristics associated with an intra-amoebal lifestyle remain largely unknown.

To date, few bacterial genomes from natural pathogens of amoebae have been fully sequenced, including those from (i) three strains of *Chlamydia*-related bacteria, i.e., "*Candidatus *Protochlamydia amoebophila" [11], *Parachlamydia acanthamoebae *[12] and *Waddlia chondrophila *[13]; (ii) five strains of *Legionella pneumophila *(Philadelphia, Lens, Paris, Corby, and Alcoy)[14-17] and two strains of *L. longbeachae *[18]; (iii) *Rickettsia bellii *[19];(iv) and a member of *Bacteroidetes*, "*Candidatus *Amoebophilus asiaticus" [10].

Recently, the new *Legionella *species *L. drancourtii*, initially named *Legionella*-like amoebal pathogen 12 (LLAP-12), was described within an *Acanthamoeba *sp. [20]. This bacterial species, known to be a strict intracellular pathogen of amoebae, exhibits a genome that is distinctly larger than its closest relatives, *Francisella tularensis *and *Coxiella burnetii *[7]. Despite its strict intracellular lifestyle, *P. acanthamoebae *exhibits a 3-fold larger genome than members of the *Chlamydia *genus [12] which includes human and animal pathogens that do not grow in amoebae [21]. These characteristics contrast with the observed genome-size reduction that is associated with the transition from a free-living to an intracellular lifestyle [22,23]. It has been proposed that the larger genome size might be linked to the sympatric lifestyle of intra-amoebal bacteria and to gene exchange among amoeba-resistant microorganisms inside the amoeba or between these microorganisms and the amoeba itself [7,24].

A major mechanism involved in genetic exchanges between amoeba-resistant bacteria and their host is the type IV secretion system (T4SS) [25,26]. This widespread system can be divided into three major subtypes: those that mediate DNA transfer (F-like system), and those that translocate proteins and nucleoprotein complexes (P- and I- like systems) [27]. All of the studied genomes of intra-amoebal bacteria, including *Legionellae, R. bellii *and *Pr. amoebophila*, exhibit a T4SS of differing subtypes [14,15,17,19,28,29], except for *W. chondrophila*, in which no such system has been identified [13]. Interestingly, a partial F-like conjugative system has been identified in the draft genome of *P. acanthamoebae *[12].

In this study, we sequence the genome of the *L. drancourtii *strain LLAP-12 and compare it to the genome of the *P. acanthamoebae *strain Hall's coccus in order to identify genes that could have been exchanged between these two amoebal pathogens and, more widely, between *Chlamydiae *and *Legionellae*.

## Results

### Dirty genomes of *L. drancourtii *and *P. acanthamoebae*

Sequencing reads from *L. drancourtii *strain LLAP-12 obtained from 454 pyrosequencing were assembled into 213 contigs that were further organized into 58 scaffolds (see Additional file 1, **Materials**). The genome had a total size of 4,062,386 bp and a G+C content of 39%. A total of 3,965 protein-coding ORFs with an average size of 869 bp were identified and cover 84.7% of the genome. The published genome of the *P. acanthamoebae *strain Hall's coccus [12] comprises 93 contigs and has a total of 2,971,261 bp with a G+C content of 38%. The 2,809 predicted genes have an average size of 943 bp and encompass 89% of the genome. The genome of *Legionella drancourtii *and *Parachlamydia acanthamoebae *have been deposited in GenBank under accession number ACUL02000000 and NZ_ACZE00000000, respectively.

### Orthologues identification

**Figure 1:**
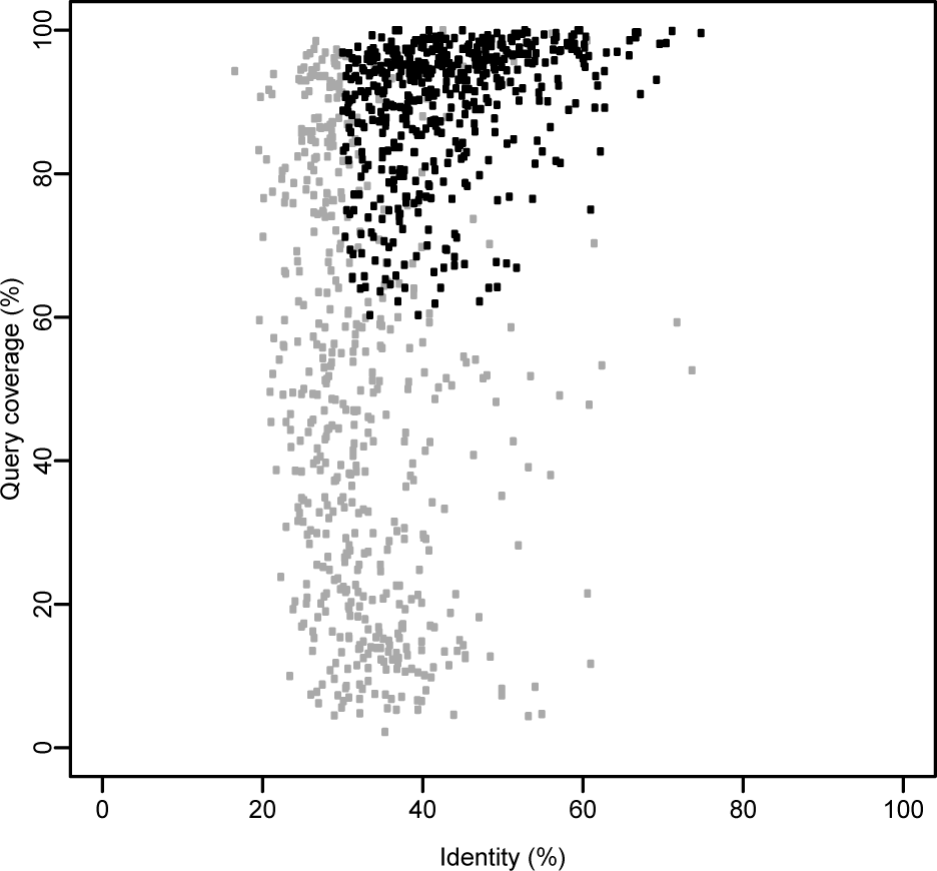
**Definition of orthologous proteins**. Representation of the identity (%) and coverage (%) of the query alignment that results from a BLASTP search of *P. acanthamoebae *versus *L. drancourtii*. Of the 1023 proteins showing reciprocal best blast hits, 508 exhibited a similarity greater than 30% and an alignment coverage longer than 60% of the query (black) and were defined as orthologues. Of the remaining 515 proteins (gray), 53 did not fulfill the same criteria in the reciprocal analysis and, therefore were discarded, although they appear on this figure as passing the cutoffs. The correlation coefficient between both of the reciprocal BLAST analyses was 0.97 and 0.80 for the percentage identity and the percentage coverage, respectively.

**Figure 2:**
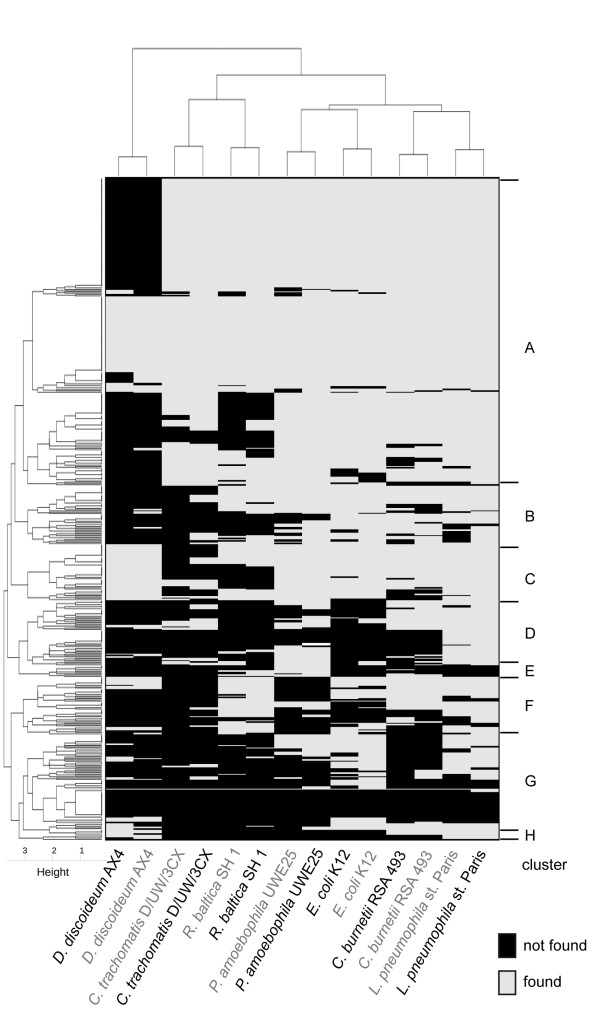
**Gene clusters**. Orthologous genes were subjected to BLASTP against seven other organisms, colored in gray and black when using *Parachlamydia *and *Legionella *proteins as queries, respectively. Genes were clustered according to their presence (gray) or absence (black) in the six related bacteria and the amoebal species, *D. discoideum*. The genes form 8 major groups at a height of 3 (A to H).

To identify orthologous genes that might have been acquired by lateral gene transfer, proteins of *P. acanthamoebae *and *L. drancourtii *were subjected to reciprocal BLASTP searches. A total of 508 orthologous proteins were identified as exhibiting a reciprocal similarity percentage > 30% and an alignment length coverage > 60% (Figure 1). Each orthologue was subjected to BLASTP homology searches against one amoebal and six bacterial genomes and was considered present if it fulfilled identical cutoffs for similarity and coverage. A clustering analysis according to the presence or absence of the orthologues in the different organisms was used to categorize the genes into eight different categories, as illustrated in the heatmap in Figure 2. The clustering of bacteria in the horizontal top tree indicates that consistent results were obtained when using *P. acanthamoebae *or *L. drancourtii *orthologues as queries against the other genomes. Cluster A consists of core genes that are present in nearly all bacteria. Clusters B and C represent core genes generally absent in *C. trachomatis *and/or *R. baltica *but respectively absent and present in the amoeba *Dictyostelium*. The absence of an ortholog in *C. trachomatis *is likely due to the genome reduction that arose in the *Chlamydia *genus, whereas the absence of an ortholog in *R. baltica *might reflect the divergence of the species. The remaining clusters are made of differently conserved genes. Genes in cluster D are mainly present in *Pr. amoebophila*, *L. pneumophila*, and in *C. burnetii*. The small cluster E contains genes mainly present in *Pr. amoebophila*. Genes in cluster F are mostly present in *L. pneumophila*, *C. burnetii *and *R. baltica *and to a lesser extend in *E. coli*. Cluster G is formed of two subsets: one subset of genes absent from all compared genomes, and another subset containing genes present in *Pr. amoebophila *and *E. coli*. Cluster H mainly represents genes encoded by the gamma-proteobacteria and the amoeba *Dictyostelium *but absent from *Chlamydiales *and Planctomycetales. The genes were classified according to the number of hits obtained against each of the six bacterial genomes used (Table 1).

**Table 1:** Orthologous gene classification

		Related to *L. drancourtii*			Related to *P. acanthamoebae*		
		
Score	No. of genes	*Legionella pneumophila*	*Coxiella burnetii*	*Escherichia coli*	*Protochlamydia amoebophila*	*Chlamydia trachomatis*	*Rhodopirellula baltica*
0	20	0	0	0	0	0	0
1	27	15	0	8	4	0	0
2	37	28	16	10	12	0	8
3	59	52	27	41	32	6	19
4	77	76	60	62	58	16	36
5	80	80	71	74	79	54	42
6	208	208	208	208	208	208	208

This table indicates the total number of proteins with a score of 0 to 6 based on their presence in the six bacteria when using *P. acanthamoebae *or *L. drancourtii *orthologous proteins as queries for the BLASTP searches. The number of corresponding genes present in each bacterium is detailed in the following columns. The bacteria in the first three columns are decreasingly related to *L. drancourtii*, andthose indicated in the last three columns exhibit diminishing relatedness to *P. acanthamoebae*.

### Conservation of orthologous genes

*Core gene identification: *Of the 508 orthologues, 208 were found in the genomes of *Pr. amoebophila*, *C. trachomatis*, *R. baltica*, *L. pneumophila*, *C. burnetii*, and *E. coli *(score 6; super-cluster A in Figure 2 and Additional file 2, **Table S1**). Of these orthologues, 92 coding sequences (44%) were considered essential genes [30] and, notably, encode for 32 ribosomal proteins and 11 tRNA synthetases. In addition, 80 genes were present in five of the six screened genomes (score 5; Additional file 3, **Table S2**), of which 13 (16%) belonged to the minimal gene set. These sequences included 9 ribosomal proteins and 3 tRNA synthetases due to their borderline values for identity and alignment coverage. Of these 80 coding sequences, 26 (33%) were absent from the *C. trachomatis *genome, reflecting the important genome reduction that has occurred in this bacterial clade, especially among genes encoding for metabolic functions (12 genes belong to COGs C, E, F, G or H). In addition, 38 genes (48%) were absent from the *R. baltica *genome, probably as a result of its greater phylogenetic distances to *Parachlamydia *and *Legionella *than the other bacteria screened. Only 9, 6, 1 and 0 genes were missing in the genomes of *C. burnetii*, *E. coli*, *Pr. amoebophila *and *L. pneumophila*, respectively. Finally, 77 genes were present in four of the six studied genomes (score 4; Additional file 4, **Table S3**) and 4 of them (5%) belonged to the minimal gene set [30]. All of the coding sequences with a score of 4 or above were considered to be common genes found in most bacteria and were therefore not further investigated for potential HGT.

*Accessory orthologues: *Of the 143 remaining genes, 59 were identified in three screened bacterial species (score 3; Additional file 5, **Table S4**). There was no obvious logical pattern of distribution among the bacteria because no genes were common to the three amoebal pathogens included in the analysis (*L. pneumophila, C. burnetii*, and *Pr. amoebophila*), and only ten coding sequences were common to *γ-proteobacteria *(*L. pneumophila*, *C. burnetii*, and *E. coli*). Moreover, bacteria belonging to the *Chlamydiae-Planctomycetes *superphylum (*Pr. amoebophila*, *C. trachomatis*, and *R. baltica*) encoded for no common gene in this category. Thirty-seven orthologues exhibited homologues in two species (score 2; Additional file 6, **Table S5**). Unsurprisingly, *L. pneumophila *contained 76% of these genes indicating its close phylogenetic distance to *L. drancourtii*. In contrast, none of these genes were found only in bacteria belonging to the *Chlamydiales *order (*Pr. amoebophila *and *C. trachomatis*), while 12 genes were only found in bacteria belonging to the *Legionellales *order (*L. pneumophila *and *C. burnetii*).

In addition, 27 genes harbored similarities to a single species (score 1; Additional file 7, **Table S6**): *L. pneumophila *(15), *E. coli *(8) or *Pr. amoebophila *(4). Finally, as visualized in Figure 2 (cluster G), 20 genes were only present in *P. acanthamoebae *and *L. drancourtii *(score 0; Additional file 8, **Table S7**). Only one of these was also present in *D. discoideum*: a conserved protein of unknown function that was also encoded only in the Corby strain of *L. pneumophila *and not in the Paris strain of *L. pneumophila *that was used in BLAST analyses. Interestingly, this category included an operon for histidine biosynthesis, which was composed of four genes (378, 379, 442, and 443), that was only found in *P. acanthamoebae *and the recently sequenced *W. chondrophila *[13] but not in the more closely-related *Pr. amoebophila*. The corresponding genes in *L. drancourtii *were encoded at two different genomic regions and the four genes exhibited divergent phylogenies that render an acquisition of the entire operon from *Legionella *implausible.

### Horizontal gene transfers

**Figure 3:**
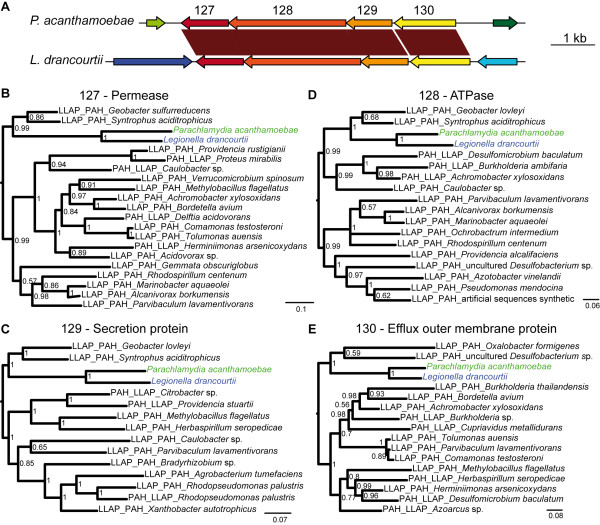
**The ABC transporter operon**. (A) An operon encoding the ABC transporter was present in both *P. acanthamoebae *and *L. drancourtii*. (B, C, D and E) A bayesian tree of each orthologous protein (ID 127, 128, 129, and 130) encoded in the operon and their 10 best BLAST hits, restricted to one hit per bacterial genus. Sequences retrieved using *L. drancourtii *or *P. acanthamoebae *as the query in a BLAST homology search are tagged with an LLAP or PAH prefix, respectively. Sequences belonging to the best BLAST hit for both orthologues harbor both prefixes. All of the proteins encoded for in the operon exhibit a similar phylogenetic tree, which shows a clustering of *P. acanthamoebae *and *L. drancourtii*, thereby supporting the hypothesis of a single transfer event for all four genes.

To identify potential gene transfers, the 143 genes with scores of 0 to 3 were subjected to phylogenetic reconstruction using the 20 best BLAST hits from each *P. acanthamoebae *and *L. drancourtii *orthologues. The topology of the trees deviated from the expected taxonomic distribution of representatives from the *Chlamydiales *and *Legionellales *orders in 37 cases that are detailed in Table 2. Overall, both *P. acanthamoebae *and *L. drancourtii *genes clustered together in 7 phylogenies (Additional file 9, **Figure S1**). Interestingly, four of these genes (ID 127, 128, 129, and 130) were organized in an operon that encodes for a permease and a membrane anchored ATP-binding protein of an ABC-2 type transporter, an HlyD family secretion protein and an outer membrane efflux protein (Figure 3). Similar systems that are widespread among gram-negative bacteria such as *E. coli, Serratia marcescens *and *Pseudomonas aeruginosa *form a specialized secretory system for proteins, such as pore-forming toxins, proteases, lipases and S-layer proteins [31]. Among the six bacteria screened, a partial operon was found only in *E. coli*, where the first three coding sequences had strong homologues, but the fourth gene encoding for the outer membrane efflux protein exhibited a homologue at a more distant locus. Notably, the tree topology was similar for all of these proteins (Figure 3) suggesting that the entire operon was exchanged between *L. drancourtii *and *P. acanthamoebae *in a single event.

**Table 2:** Potential horizontal gene transfers identified

Score	*PAH-LLAP*	*PAH-Legionellales*	*Legionellales-Chlamydiales*	*PAH/LLAP-Rickettsiales*	*PAH/LLAP*-Others	Gene product	COG accession	COG group
**0**	101					Hypothetical protein		
	158					Choloylglycine hydrolase	COG3049	M
		191		191 *PAH-Rickettsiales*		Acetyltransferase	COG0454	KR
		192		192 *PAH-Wolbachia*		Aminoglycoside phosphotransferase family protein	COG3173	R
	473					putative phosphotransferase	COG2334	R
		475				Hypothetical protein	COG0500	QR
		492				Toluene efflux pump outer membrane protein	COG1538	MU
**1**		96				Conserved hypothetical protein	COG0599	S
	127					ABC-type multidrug transporter, permease	COG0842	V
	128					ABC-type multidrug transporter, ATP-binding protein	COG1131	V
	129					HlyD family secretion protein	COG0845	M
				243 *LLAP-Rickettsia*		Beta-lactamase	COG2602	V
		246				Acetyltransferase, GNAT family	COG0454	KR
		247				Hypothetical protein	COG2132	Q
			282			Conserved hypothetical protein	COG4804	S
		387		387 *PAH-Legionellales-Orientia*		Hypothetical protein	COG0500	QR
**2**		11				Succinylarginine dihydrolase	COG3724	E
		86				Metallo-beta-lactamase family protein	COG1234	R
		166				Multidrug resistance protein	COG0477	GEPR
			207		207 *LLAP*-*Naegleria*	7-dehydrocholesterol reductase	COG2020	O
		301				Acyl-CoA dehydrogenase	COG1960	I
				388 *LLAP-Rickettsia*		Aminoglycoside N(6')-acetyltransferase	COG1670	J
		400*				Putative transcriptional regulator	COG0583	K
		421				Putative outer membrane efflux protein	COG1538	MU
					458 *Chlamydiales-*Eukaryotes	Alpha keto acid dehydrogenase complex E1 component alpha subunit	COG1071	C
		474				DNA protection during starvation protein	COG0783	P
**3**			90			Multidrug resistance protein B homolog	COG0477	GEPR
	130					Solvent efflux pump outer membrane protein	COG1538	MU
				134 *Chlamydiales-Ehrlichia/Wolbachia*		Proton/sodium-glutamate symport protein	COG1301	C
					208 *LLAP-A.asiaticus*	PEBP family protein	COG1881	R
				211 *PAH-Rickettsia*		Putative 6-pyruvoyl tetrahydrobiopterin synthase	COG0720	H
					240 *LLAP-*Eukaryotes	Hypothetical protein	COG1092	R
			263	263 *LLAP-Rickettsia*		DNA protection during starvation protein 2	COG0783	P
				390 *Legionellales-Rickettsiales*		ABC-type transporter, permease and ATPase subunit	COG1132	V
		413				Cation-transporting ATPase	COG2217	P
		422*				Inner membrane transport permease YbhR	COG0842	V
		466*				Methylisocitrate lyase	COG2513	G

* clustering with *Legionella pneumophila *only, *PAH *= *Parachlamydia acanthamoebae, LLAP *= *Legionella drancourtii*

This table presents the classification by score and ID numbers of the *L. drancourtii *and *P. acanthamoebae *orthologues that exhibit a phylogenetic reconstruction suggesting horizontal gene transfer events. The gene product annotation, COG accession number and COG group are indicated for each gene.

*L. drancourtii *never clustered with several members of the *Chlamydiales *order. However, *P. acanthamoebae *branched with the *Legionellales *clade in 10% of the trees (15/143) (Additional file 10, **Figure S2 A-O**). A good example of this clustering is a hypothetical protein (ID 247), where *P. acanthamoebae *clustered with a branch containing *Francisella *and two *Legionella *species, suggesting that a transfer occurred before the divergence of the *Legionella *and *Francisella *genera. In three additional instances (ID 400, 422, and 466), *P. acanthamoebae *clustered specifically with *L. pneumophila *and far from other *Legionellales *(Additional file 10, **Figure S2 P-R**) suggesting a transfer between these two species. These genes were located on different contigs and encoded a permease, an HlyD family protein belonging to an ABC-transporter, and a putative transcriptional regulator. An example of a protein wrongly considered only to be present in *L. drancourtii *and *P. acanthamoebae *is an acetyltransferase (ID 191) that has a borderline similarity value of 29.4% with *L. pneumophila*. In fact, the corresponding gene was found in several members of the *Rickettsiales *order (*Rickettsia, Wolbachia*, and *Orientia*), in *Legionella *species and in *P. acanthamoebae*. We hypothesize that this protein's presence in *Parachlamydia *results from a HGT with an ancestor of the *Legionellales *order.

**Figure 4:**
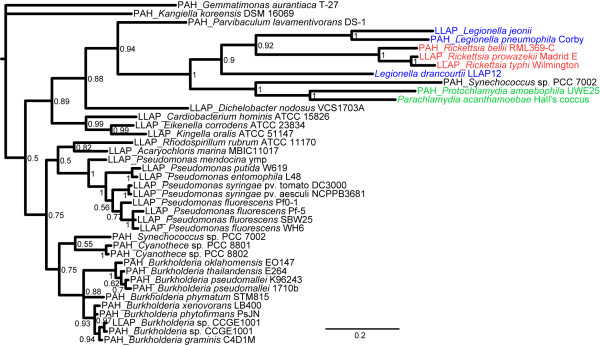
***dpsA *phylogeny**. The bayesian tree for *dpsA *(ID 263), a protein involved in protection against oxidative stress, and the 20 best BLAST hits shows the clustering of proteins from *Rickettsia *with *Legionella *species and, to a lesser extent, with *Parachlamydiaceae *and *Synechococcus*, a cyanobacterium. Sequences retrieved using *L. drancourtii *or *P. acanthamoebae *as the query in a BLAST homology search are tagged with an LLAP or PAH prefix, respectively. Sequences belonging to the best BLAST hits for both orthologues harbor both prefixes.

Furthermore, the *Legionellales *and *Chlamydiales *orders were directly related in the tree that was inferred from four genes (ID 90, 207, 263, 282) that encode for a multidrug resistance protein, a 7-dehyrocholesterol reductase, a protein that protects DNA during starvation and a conserved hypothetical protein, respectively (Additional file 11, **Figure S3**). The gene involved in the protection against oxidative stress (*dpsA*, ID 263) presented a particular topology. A phylogenetic tree inferred from an analysis with the 20 best BLAST hits indicated that this gene was recently exchanged between *Rickettsiales *and *Legionellales *and that an exchange between *Legionellae *and *Chlamydiae *had occurred earlier (Figure 4). Notably, two different copies of this gene were also present in the cyanobacterium *Synechococcus*, one of which branched closely to *Chlamydiales*, suggesting an exchange with an ancestral *Chlamydiae*, and another copy clustered with other cyanobacterial genomes and β*-proteobacteria*.

**Figure 5:**
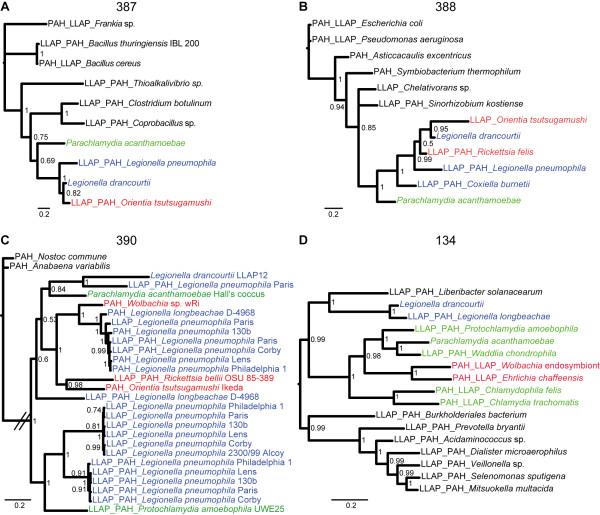
**Potential gene transfers with members of the *Rickettsiales *order**. The bayesian trees for each orthologous gene (ID 387, 388, 390, and 134) and their 10 best BLAST hits show the clustering of *Chlamydiales *and *Legionellales *with members of the *Rickettsiales *order. The retrieval of BLAST hits was restricted to one per bacterial genus for (A), (B), and (D) but not for (C). Sequences retrieved using *L. drancourtii *or *P. acanthamoebae *as the query in a BLAST homology search are tagged with an LLAP or PAH prefix, respectively. Sequences belonging to the best BLAST hits for both orthologues harbor both prefixes.

The phylogenetic analysis identified 9 cases of potential gene transfer between *P. acanthamoebae *or *L. drancourtii *and *Rickettsia, Orientia, Wolbachia *and *Ehrlichia *(Additional file 12, **Figure S4**). Of these cases, five genes (ID 243, 263, 387, 388, and 390) may have been horizontally transferred between *L. drancourtii *and *Rickettsia*, including a beta-lactamase (ID 243). The analysis of the ID 387-encoded hypothetical protein clustered *P. acanthamoebae, Legionellales *and *Orientia *together. The close relationship between *L. drancourtii *and *Orientia *suggests a transfer to the latter species (Figure 5). The tree of the aminoglycoside transferase (ID 388) illustrates a complex situation where all of the intracellular bacteria were grouped in a single cluster, suggesting a common ancestral source for this gene. The ATPase and permease subunits of an ABC-type transporter (ID 390) exhibited the remarkable phylogenetic pattern of being nearly exclusive to intracellular species. However, this tree exhibited an atypical configuration that showed no monophyly among *Legionella *and *Parachlamydiaceae*. Indeed, *L. drancourtii *clustered with *L. pneumophila *str. Paris and *Parachlamydia*, whereas another gene from *L. pneumophila *clustered with *Protochlamydia*. This topology suggests the presence of multiple paralogues within the *L. pneumophila *genome. We hypothesize that duplication events first occurred in *L. pneumophila *and that one paralogue was subsequently horizontally transferred to *Protochlamydia *and another paralogue to *Parachlamydia*. Finally, the tree for a proton/sodium-glutamate symport protein (ID 134) suggests an exchange between *Chlamydiales *and an ancestral *Rickettsiales *(Figure 5).

In three cases (ID 207, 240 and 458), similarity searches retrieved eukaryotic proteins matching *L. drancourtii *and *P. acanthamoebae *proteins, including a 7-dehydrocholesterol reductase and a conserved hypothetical protein (ID 207 and 240, respectively) from *L. drancourtii *that seem closer to eukaryotes than to bacteria with the exception of *L. longbeachae *and a pyruvate dehydrogenase E1 alpha subunit (ID 458) (Additional file 13, **Figure S5)**. Interestingly, BLAST searches with the 7-dehydrocholesterol reductase retrieved a protein from the amoeba *Naegleria gruberi *that clusters with *L. drancourtii *in the phylogenetic tree. In a recent study, this gene was proposed to have been acquired from viridiplantae [32], but this earlier phylogenetic reconstruction did not include *Naegleria*. Finally, two widely conserved enzymes (ID 288 and 289, respectively) that encode for a malate synthase and an isocitrate lyase are syntenic in both genomes. A phylogenetic reconstruction with their twenty best BLAST hits and amoebae showed that the malate synthase of *L. drancourtii *clustered with *Dictyostelium discoideum*, suggesting a gene exchange from a *Legionella *ancestor to amoebae, as previously reported [33].

The genomes of *L. drancourtii *and *P. acanthamoebae *harbor respectively 45 and 8 insertion sequences (ISs), a number probably underestimated due to the likely presence of such elements in gaps of the genomic sequence. One IS of type ISNpu13 is located 2000 bp upstream the gene LGD_5795 (ID 246) that encodes an acetyltransferase. There were no ISs close to other genes identified as potentially acquired by HGT (Table 2).

The genomic nucleotide composition, notably the GC content and codon usage, varies widely among the organisms. As a consequence, regions that were exchanged recently present an imprinting with the donor's characteristics. However, mutational pressures tend to adapt the compositional features of foreign DNA to that of its host over time, thus making the transferred region indistinguishable [34]. In our analysis, the GC content of the 37 genes suspected to have been acquired by HGT based on phylogeny did not show a marked difference in the GC content compared to the rest of the *Parachlamydia *or *Legionella *genes (Additional file 14, **Figure S6**). The mean GC content for *Parachlamydia *and *Legionella *is respectively 38.5% and 39% for all genes and, more precisely, 33.7% and 32.6% at the third codon position, which is less constrained for the conservation of amino acid properties. An interesting case was the operon that encodes an ABC transporter that showed the highest GC content, exhibiting values between 41% and 45% in *P. acanthamoebae*. In contrast, the GC content of this operon in *L. drancourtii *was less distinguishable from the mean gene GC values. This finding suggests that *P. acanthamoebae *acquired this operon by HGT with a partner that has a higher GC content than *L. drancourtii*.

Most exchanged genes encode poorly characterized functions represented by COG category R. However, several genes that belong to the overrepresented categories R, V, M and P encode putative functions linked to the transport of various molecules and resistance to toxic compounds.

## Discussion

In this study, the genes in the draft genomes of *P. acanthamoebae *and *L. drancourtii *were compared; we identified 508 orthologues, which were subsequently searched for in increasingly distantly related organisms. Although limited by the small number of related organisms that we used to define conserved core genes and less-conserved genes, our approach allowed us efficiently to discard 72% of the orthologues that are widespread within bacteria. The remaining 28% of the genes were submitted to phylogenetic reconstructions, which enabled us to detect 7 putative HGTs between *P. acanthamoebae *and *L. drancourtii*. In addition, 18 genes were potentially transferred from *Legionellales *to *P. acanthamoebae*, whereas no genes were transferred from *Chlamydiales *to *L. drancourtii*. As expected, half of these potential HGTs encode for transporters and resistance mechanisms that could be of particular importance for the bacteria to resist to the microbicidal defences of its amoebal host and to survive with high concentrations of toxic compounds. The remaining HGTs encode for metabolic functions or members of poorly characterized protein families. Their importance and their role regarding the amoebal pathogenicity of *L. drancourtii *and *P. acanthamoebae *remain to be determined.

We acknowledge the fact that the stringent reciprocal identity and coverage cut-offs constitute one major limitation of this method, as they may falsely classify some genes as absent from one of the selected related species when they are, in fact, present. Genes that were probably acquired by HGT showed a large distribution in their percentage of identity, ranging from 30% to 71%, but they all exhibited an alignment coverage greater than 80% indicating that a more stringent criterion could have been used (Additional file 15, **Figure S7**). The phylogenetic reconstructions enabled us to pinpoint such cases because closely related sequences were retrieved by BLAST searches. One major cause of these false negatives are borderline values in one of the variables, as highlighted for the previously mentioned acetyltransferase (ID 191), in which the percentage identity was 29.3%, and the coverage 81%.

Another bias may stem from the sequencing technology (454/Roche Genome Sequencer) and the gene prediction method used for both genomes; other approaches may have been used in the other six genomes screened. A particular example is the case of two orthologues (ID 48 and 49) that correspond to a single gene that was artificially split in two different coding sequences. After manual visualization of the sequencing data from both genomes, it appeared that frameshifts were caused by two homopolymer errors due to the sequencing technology at similar positions in the two bacteria. This artificially split gene encodes an ATP-dependent Clp protease (ClpB) that is, in fact, present in three species: *L. pneumophila, C. trachomatis *and *Pr. amoebophila*, although it was considered to be absent from the six genomes screened.

In addition, a number of proteins that are considered orthologues in *L. drancourtii *and *P. acanthamoebae *do not share a common history. In these cases, BLAST searches retrieved proteins from completely different organisms and the phylogenies clearly showed a divergent history, with the *Parachlamydia *and *Legionella *hits being grouped in distinct clusters (e.g., ID 301, Additional file 10, **Figure S2**). The phylogenies exhibiting this topology are easily identifiable and, therefore, do not cause a bias in the results. One option to avoid misidentifying orthologues would have been to perform a BLAST comparison versus the non-redundant database and to assign orthology only if the gene occurred within the best BLAST hits.

We did not identify any gene transfers from *Chlamydiales *to *L. drancourtii*. However, this finding may be due to the limited number of genomic sequences from *Chlamydia*-related bacteria available in the non-redundant database. Indeed, most published genomes belong to the family *Chlamydiaceae*, which includes bacteria that are highly adapted to their non-amoebal eukaryotic hosts and exhibit small genomes that typically range from 1 Mbp to 1.2 Mbp. Additionally, the *Chlamydiales *order presents a biphasic developmental cycle the relatively inactive extracellular form is surrounded by a highly cross-linked extracellular matrix [35] that might relatively non-permissive to genetic exchanges. The replicating form, possessing a larger size and reduced extracellular matrix, might be more permeable to such exchanges that should subsequently arise within the host inclusion. The ability of *Chlamydia*-related bacteria to grow within amoebae would thus facilitate gene exchanges with other amoeba-infecting microorganisms. This hypothesis would be strengthened by *in vitro *evidence of co-localisation in amoebae of *P. acanthamoebae*, or any other amoeba-resistant chlamydiae, with *Legionella*.

Ogata *et al*. [19] suggested that gene exchanges among amoeba-resistant bacteria are an important mechanism for triggering the evolution of intracellular microorganisms. The recent analysis of the *Amoebophilus asiaticus *genome identified a large prevalence of gene transfer events among amoeba-resistant bacteria [10]. In our study, several phylogenies indicated possible HGTs between amoeba-associated bacteria (*Legionella *and *Parachlamydia*) and arthropod-associated bacteria, such as *Rickettsia, Orientia, Wolbachia*, and *Ehrlichia*. The presence of these genes in insect and arthropod endosymbionts suggests the possibility of intra-insect transfer because the *Chlamydiales *order also comprises the genera *Fritschea *and *Rhabdochlamydia*, which feature members that can infect arthropods [36-39]. Thus, as proposed by Thomas *et al*. [40], gene exchanges might be a common mechanism in intracellular bacteria that exhibit a sympatric lifestyle and may occur not only between amoebal symbionts but also between those found in arthropods. In addition, we identified a significant BLAST match with Mimivirus, a large amoeba-infecting virus, for a hypothetical protein (ID 404), thereby supporting the hypothesis that intra-amoebal viruses are also capable of genetic exchanges with other microorganisms found in this environment [7].

In most cases, the *Legionella *or *Parachlamydia *proteins were largely diverged from their closest neighbors, suggesting that the gene transfers were evolutionarily ancient events. The inclusion of the best BLAST hits vs NR in the phylogenetic reconstruction identified the putative partner of transfer among all available sequenced genomes. As an example, the phylogenetic reconstruction of the PEBP family protein (ID:208) clustered *L. drancourtii *with the amoebal symbiont *Amoebophilus asiaticus *(Table 2). Due to the lack of sequenced genomes in species closely-related to *L. drancourtii *or *P. acanthamoebae*, the direction of the transfer and the period of exchange relative to bacterial speciation cannot be unequivocally established. There is no strong evidence for recent HGT events that can enable us to pinpoint the exact partners of the transfer, and an intermediate host that has not been sequenced yet might be implicated in the future.

No genes were surrounded on both sides by IS but, since no recent HGT was identified, insertion sequences might have diverged over time sufficiently to impair their identification. The close GC content values between the *L. drancourtii *and *P. acanthamoebae *species and the large divergence in the GC content among genes prevent us from confirming gene exchanges between these two partners by this method. Moreover, the large range of GC content within a bacterial order makes the use of this measure difficult for discriminating a potential origin of transfer because the phylogenetic tree usually shows distant relationships between our candidate partners.

## Conclusion

This study presents a first genome-scale analysis that provides evidence for numerous HGT that occurred among three major intra-amoebal bacterial phyla. Moreover, this article reports a new genome of an amoebal pathogen, *L. drancourtii*, that opens new possibilities for the investigation of such important horizontal transfer events and provides data for researchers interested in the biology of this species. With the availability of additional genome sequences, a complete study of all sequenced intra-amoebal genomes would be of large interest and will bring in the future a more complete picture of gene exchanges occurring between amoeba-resistant microorganisms. As previously suggested [7], the amoebae probably have a major role in bringing amoeba-associated microorganisms close enough to facilitate genetic exchanges. The occurrence of HGT between intra-amoebal pathogens is of importance, since such gene exchanges may play an important role in the selection of bacteria able to resist both the amoebae and other phagocytic cells such as the macrophages, which thus represent good candidates as new pathogens

## Methods

### Study design

To study the occurrence of gene transfer between amoebal pathogens, we sequenced the genome of the *L. drancourtii *strain LLAP-12 and compared it to the draft genome of the *P. acanthamoebae *strain Hall's coccus. Details on the dirty genome sequencing method used for *L. drancourtii *are provided with the Supplementary Materials (Additional file 1). After identifying orthologous genes present in both the *L. drancourtii *and *P. acanthamoebae *genomes, we searched for homologous genes in six bacterial genomes and one amoebal genome, as detailed below. The presence or absence of every orthologue in each clade was used to infer a heat map and a score. The corresponding clusters were investigated in more details. In addition, selected genes were compared by BLAST [41] to the non-redundant (nr) GenBank database and subjected to phylogenetic analyses.

### Identification of conserved genes

To identify genes that were present in both *L. drancourtii *and *P. acanthamoebae*, we performed BLASTP comparisons between both genomes and the best reciprocal hits were retrieved if they exhibited a similarity > 30% and a query and hit length coverage > 60%. These conserved genes were searched for in one amoebal genome (*Dictyostelium discoideum *AX4 [NC_007087-NC_007092], the only fully sequenced amoebal genome available to date) and six bacterial genomes with a decreasing degree of phylogenetic relatedness respectively to *L. drancourtii *and *P. acanthamoebae*. Model organisms or medically important bacteria were selected within the same family (*Legionella pneumophila *Paris [NC_006368], respectively *Pr. amoebophila *UWE25 [NC_005861]), within the same order (*Coxiella burnetii *RSA 493 [NC_002971], respectively *Chlamydia trachomatis *D/UW-3/CX [NC_000117]) and within the same phylum (*Escherichia coli *K12 substr. MG1655 [NC_000913.2], respectively *Rhodopirellula baltica *SH1 [NC_005027]). Each gene was considered present if it exhibited > 30% identity and had a length coverage > 60% on both the query and hit. The genes were classified according to the number of hits obtained using *P. acanthamoebae *or *L. drancourtii *as the query against each of the six bacterial genomes based on a score of 0 (no hits in any f the screened genomes) to 6 (hits in all of the screened bacterial genomes).

### Gene clustering and the definition of core genes

Hierarchical clustering was performed based on the Euclidean distance of the presence or absence of each gene in the studied genomes. Using the H-clust function (an in-house R script), super clusters were defined at a dendrogram height equal to 3. The clusters were represented on a heat map drawn using an in-house R script [42]. Core genes were defined as any gene present in at least 4 of the 6 screened genomes (score ≥ 4) and were not further analyzed.

### BLAST and phylogenetic analyses

A BLASTP search against the nr database http://blast.ncbi.nlm.nih.gov/Blast.cgi was performed for genes with scores between 0 and 3. For each gene, a phylogenetic analysis was conducted with the 20 best BLASTP hits of *P. acanthamoebae *and *L. drancourtii *that was restricted to one representative species per genus. Sequence alignment was performed using Muscle [43]. Phylogenetic relationships among species were inferred with PhyML (PHYlogenetic inferences using Maximum Likelihood) [44] using the WAG model. For particularly interesting cases, a phylogeny based on a Bayesian inference was performed using MrBayes [45] on alignments curated by Gblocks [46].

### Identification of lateral gene transfer

Genes exhibiting a topology different from that expected for core genes were classified according to one of the five following topologies: i) *P. acanthamoebae *genes clustering with *L. drancourtii*; ii) *P. acanthamoebae *genes clustering with *Legionellales*; iii) *Legionellales *genes clustering with *Chlamydiales*; iv) *P. acanthamoebae *and/or *L. drancourtii *genes clustering with *Rickettsia, Ehrlichia *and *Wolbachia *and (v) genes matching eukaryotic proteins by BLAST homology searches.

To characterize further the genes exhibiting an unexpected phylogenetic topology, their GC content was examined relative to the genomic mean with R [42] using the seqinR package [47]. A BLASTN comparison was achieved versus ISfinder [48] with an e-value of 1e-5 to search for insertion sequences in the genomes of *L. drancourtii *and *P. acanthamoebae*.

## List of Abbreviations

IS: insertion sequence; HGT: horizontal gene transfer; LLAP: *Legionella*-like amoebal pathogen 12; NR: non-redundant; ORF: Open Reading Frame; PAH: *Parachlamydia acanthamoebae *strain Hall's coccus; PhyML: PHYlogenetic inferences using Maximum Likelihood; T4SS: Type IV secretion system.

## Competing interests

The authors declare that they have no competing interests.

## Authors' contributions

PEF and GGr conceived and designed the study. DR participated in the study design. GGi and CB performed the sequence comparison and phylogenetic analyses. CM made the LLAP genome draft annotation. CR performed the genome sequencing. GGi, CB, PEF and GGr analyzed the data and wrote the manuscript. All authors revised the final version of the manuscript.

## Supplementary Material

Additional file 1 **Supplemental data**. Click here for file

Additional file 2 **Table S1. Conserved orthologous genes**. This table lists the 208 orthologues of *L. drancourtii *(LLAP) and *Pr. acanthamoebae *(PAH) also found in the genomes of *Pr. amoebophila, C. trachomatis, R. baltica, L. pneumophila, C. burnetii, *and *E. coli*. Click here for file

Additional file 3 **Table S2. Orthologous genes identified in 5 bacteria**. This table presents the 80 orthologues of *L. drancourtii *(llap) and *P. acanthamoebae *(pah) identified in five among the six bacterial genomes screened (*Pr. amoebophila, C. trachomatis, R. baltica, L. pneumophila, C. burnetii, and E. coli*). The last column indicates the bacterial genome where the corresponding gene could not be identified. Click here for file

Additional file 4 **Table S3. Orthologous genes identified in four bacteria**. Accession number and annotation of the 77 orthologues of *L. drancourtii *(LLAP) and *P. acanthamoebae *(PAH) also identified in four of the six bacterial genomes screened (*Pr. amoebophila, C. trachomatis, R. baltica, L. pneumophila, C. burnetii, and E. coli)* Click here for file

Additional file 5 **Table S4. Orthologous genes identified in three bacteria**. Accession number and annotation of the 59 orthologues of *L. drancourtii *(LLAP) and *P. acanthamoebae *(PAH) also identified in three of the six bacterial genomes screened (*Pr. amoebophila, C. trachomatis, R. baltica, L. pneumophila, C. burnetii, and E. coli*). Click here for file

Additional file 6 **Table S5. Orthologous genes identified in two bacteria**. Accession number and annotation of the 37 orthologues of *L. drancourtii *(LLAP) and *P. acanthamoebae *(PAH) also detected in two among the six bacterial genomes screened (*Pr. amoebophila, C. trachomatis, R. baltica, L. pneumophila, C. burnetii, and E. coli*). The last column indicates the two bacterial species that harbour the corresponding homologous gene. Click here for file

Additional file 7 **Table S6. Orthologous genes identified in one single bacterium**. Accession number and annotation of the 27 orthologues of *L. drancourtii *(llap) and *P. acanthamoebae *(pah) identified in only one of the six bacterial genomes screened (*Pr. amoebophila, C. trachomatis, R. baltica, L. pneumophila, C. burnetii, and E. coli*). The last column indicates the bacterial species that harbours the corresponding homologous gene. Click here for file

Additional file 8 **Table S7. Orthologous genes not identified in any bacteria screened**. Accession number and annotation of the 20 orthologues of *L. drancourtii *(LLAP) and *P. acanthamoebae *(PAH) that could not be detected in any of the six bacterial genomes screened (*Pr. amoebophila, C. trachomatis, R. baltica, L. pneumophila, C. burnetii, and E. coli*). Click here for file

Additional file 9 **Figure S1. Phylogenetic trees clustering *L. drancourtii *and *P. acanthamoebae***. Maximum likelihood trees of *L. drancourtii *and *P. acanthamoebae *orthologous proteins and their 20 best blast hits, restricted to one representative per genus. In these phylogenetic reconstruction, *L. drancourtii *and *P. acanthamoebae *cluster together. Sequences retrieved by using *L. drancourtii *or *P. acanthamoebae *protein as a query, are indicated with the prefix LLAP or PAH, respectively. Bacteria belonging to the *Legionellales, Chlamydiales *and *Rickettsiales *are shown respectively in blue, green and red. Click here for file

Additional file 10 **Figure S2. Phylogenetic trees clustering *P. acanthamoebae *and *Legionellales***. Maximum likelihood trees of *L. drancourtii *and *P. acanthamoebae *orthologous proteins and their 20 best blast hits, restricted to one representative per genus. (A-O) *P. acanthamoebae *directly branches with Legionellales and (O-R) *P. acanthamoebae *clusters with *L. pneumophila *but more distantly to other Legionellales. Sequences retrieved by using *L. drancourtii *or *P. acanthamoebae *protein as a query, are indicated with the prefix LLAP or PAH, respectively. Bacteria belonging to the *Legionellales, Chlamydiales *and *Rickettsiales *are shown respectively in blue, green and red. Click here for file

Additional file 11 **Figure S3. Phylogenetic trees clustering *Chlamydiales *and *Legionellales***. Maximum likelihood trees of *L. drancourtii *and *P. acanthamoebae *orthologous proteins and their 20 best blast hits, restricted to one representative per genus, where bacteria of the *Chlamydiales *order and the *Legionellales *are directy related. Sequences retrieved by using *L. drancourtii *or *P.acanthamoebae *protein as a query, are indicated with the prefix LLAP or PAH, respectively. Bacteria belonging to the *Legionellales, Chlamydiales *and *Rickettsiales *orders are shown respectively in blue, green and red. Click here for file

Additional file 12 **Figure S4. Phylogenetic trees clustering *L. drancourtii *or *P. acanthamoebae *with *Rickettsiales *and *A. asiaticus***. Maximum likelihood trees of *L. drancourtii *and *P. acanthamoebae *orthologous proteins and their 20 best blast hits, restricted to one representative per genus, where *Chlamydiales *or *Legionellales *representatives are directy related to other intracellular bacteria such as *Rickettsia, Ehrlichia, Orientia *or *Wolbachia*. In the last phylogenetic reconstruction *Legionella *clusters with another intra-amoebal bacterium, *Amoebophilus asiaticus*. Trees for ID 191, 192 and 387 are found in Additional file 2, Figure S1 and the tree for ID 263 is shown in Additional file 4, Figure S3. Sequences retrieved by using *L. drancourtii *or *P. acanthamoebae *protein as a query, are indicated with the prefix LLAP or PAH, respectively. Bacteria belonging to the *Legionellales, Chlamydiales *and *Rickettsiales *orders are shown respectively in blue, green and red. Click here for file

Additional file 13 **Figure S5. Phylogenetic trees with eukaryotic representatives**. In two cases (A and C), eukaryotic sequences were identified by BLASTP homology searches. In another tree (B), *L. drancourtii *clustered with the amoeba-associated *Bacteroidetes *named *Amoebophilus asiaticus*. Maximum likelihood trees of *L. drancourtii *and *P. acanthamoebae *orthologous proteins were build using their 20 best blast hits, restricted to one representative per genus. Sequences retrieved by using *L. drancourtii *or *P. acanthamoebae *protein as a query, are indicated with the prefix LLAP or PAH, respectively. Bacteria belonging to the *Legionellales, Chlamydiales *and *Rickettsiales *orders are shown respectively in blue, green and red. Click here for file

Additional file 14 **Figure S6. Genic GC content**. The genic GC contents of *P. acanthamoebae *(A) and *L. drancourtii *(B) are shown in grey, whereas orthologous genes are shown in light pink. Horizontally transferred genes are colored by categories of the putative partners according to the legend within the figure: in blue-green between *L. drancourtii *and *P. acanthamoebae*; in purple between *Legionellales *and *Chlamydiales *members; in pink between *L. drancourtii *or *P. acanthamoebae *and *Rickettsiales*; in yellow with Eukaryotes or *A. asiaticus*. Panels (C) and (D) present the genic GC content at the 3rd position of the codon respectively in *P. acanthamoebae *and *L. drancourtii *using a similar color-code. Click here for file

Additional file 15 **Figure S7. Percentage identity and coverage in *L. drancourtii *and *P. acanthamoebae *orthologues**. Proteins potentially transferred horizontally are colored according to four categories of gene transfer: in blue-green between *L. drancourtii *and *P. acanthamoebae*; in purple between *Legionellales *and *Chlamydiales *members; in pink between *L. drancourtii *or *P. acanthamoebae *and *Rickettsiales*; in yellow with Eukaryotes or *A. asiaticus*. Click here for file
