# Supplementary material for: Insight into cross-talk between intra-amoebal pathogens
Source: BMC Genomics. 2011 Nov 2;12:542. doi: 10.1186/1471-2164-12-542 (PMC3220658; doi:10.1186/1471-2164-12-542)
Supplement: Additional file 1 — Supplemental data. [file 1471-2164-12-542-S1.DOC]

**SUPPLEMENTARY DATA:**

SUPPLEMENTARY METHODS:

**DNA preparation**

*L. drancourtii* was grown on BCYE agar (BioMerieux, Marcy l’Etoile, France) in a 5% CO_2_ atmosphere at 32°C for 3 days. Genomic DNA was extracted using the phenol/chloroform method as previously described .

**Genome sequencing and assembly**

A four-fold genome sequencing of *L. drancourtii* was performed by pyrosequencing using the Genome Sequencer FLX (454 Life Sciences, Branford, CT) . Resulting sequence reads were assembled into consensus sequences (contigs) using the Newbler Assembler Software (454 Life Sciences, Branford, CT).

*Paired end sequencing*

A library of paired end fragments was created following the manufacturer’s instructions (454 Life Sciences, Branford, CT). This library was sequenced using the GSFLX sequencer (454 Life Sciences, Branford, CT) and resulting paired sequences were assembled with previous sequences using the Newbler Assembler Software (454 Life Sciences, Branford, CT). The assembly was verified using the CLC Genomics software (CLC Bio, Massachusetts, USA).

*Genome closing*

Sequences of gaps obtained between organized contigs were amplified by PCR using the HotStarTaq DNA Polymerase kit. PCR products were purified using NucleoFAST plates (Machery-Nagel, Hoerdt, France) and resuspended in 50 µl of sterile water. Purified products were sequenced using the BigDye® Terminator v3.1 Ready Reaction Mix (Applied Biosystems) as recommended by the manufacturer in a 3130*xl* Genetic Analyzer (Applied Biosystems).

PCR product sequences were assembled with whole genome shotgun sequences using the Newbler Assembler Software (454 Life Sciences, Branford, CT). The assembly was verified using the CLC Genomics software (CLC Bio, Massachusetts, USA). The GenBank (http://www.ncbi.nlm.nih.gov/Genbank) accession number for the *L. drancourtii* genomic sequences is ACUL02000000.

**Genome annotation**

ORF prediction on the final sequences was performed using Glimmer. ORFs were then annotated by sequence similarity using BLASTX against GeneBank (National Center for Biotechnology Information, NCBI). Genes encoding tRNA were identified with tRNAscan-SE and other RNAs were located using BLASTN.

REFERENCES

1. Fournier PE, El Karkouri K, Leroy Q, Robert C, Giumelli B, Renesto P, Socolovschi C, Parola P, Audic S, Raoult D: **Analysis of the Rickettsia africae genome reveals that virulence acquisition in Rickettsia species may be explained by genome reduction**. *BMC Genomics* 2009, **10**:166.

2. Margulies M, Egholm M, Altman WE, Attiya S, Bader JS, Bemben LA, Berka J, Braverman MS, Chen YJ, Chen ZT *et al*: **Genome sequencing in microfabricated high-density picolitre reactors**. *Nature* 2005, **437**(7057):376-380.

3. Lowe TM, Eddy SR: **tRNAscan-SE: a program for improved detection of transfer RNA genes in genomic sequence**. *Nucleic Acids Res* 1997, **25**(5):955-964.

SUPPLEMENTARY FIGURE LEGENDS

**Supplementary Figure 1. Phylogenetic trees clustering *L. drancourtii* and *P. acanthamoebae***

Maximum likelihood trees of *L. drancourtii* and *P. acanthamoebae* orthologous proteins and their 20 best blast hits, restricted to one representative per genus. In these phylogenetic reconstruction, *L. drancourtii* and *P. acanthamoebae* cluster together. Sequences retrieved by using *L. drancourtii* or *P. acanthamoebae* protein as a query, are indicated with the prefix LLAP or PAH, respectively. Bacteria belonging to the *Legionellales, Chlamydiales* and *Rickettsiales* are shown respectively in blue, green and red.

**Supplementary Figure 2. Phylogenetic trees clustering *P. acanthamoebae* and *Legionellales***

Maximum likelihood trees of *L. drancourtii* and *P. acanthamoebae* orthologous proteins and their 20 best blast hits, restricted to one representative per genus. (A-O) *P. acanthamoebae* directly branches with *Legionellales* and (O-R) *P. acanthamoebae* clusters with *L. pneumophila* but more distantly to other *Legionellales*. Sequences retrieved by using *L. drancourtii* or *P. acanthamoebae* protein as a query, are indicated with the prefix LLAP or PAH, respectively. Bacteria belonging to the *Legionellales, Chlamydiales* and *Rickettsiales* are shown respectively in blue, green and red.

**Supplementary Figure 3. Phylogenetic trees clustering *Chlamydiales* and *Legionellales***

Maximum likelihood trees of *L. drancourtii* and *P. acanthamoebae* orthologous proteins and their 20 best blast hits, restricted to one representative per genus, where bacteria of the *Chlamydiales* order and the *Legionellales* are directy related. Sequences retrieved by using *L. drancourtii* or *P.acanthamoebae* protein as a query, are indicated with the prefix LLAP or PAH, respectively. Bacteria belonging to the *Legionellales, Chlamydiales* and *Rickettsiales* orders are shown respectively in blue, green and red.

**Supplementary Figure 4. Phylogenetic trees clustering *L. drancourtii* or *P. acanthamoebae* with *Rickettsiales* and *A. asiaticus***

Maximum likelihood trees of *L. drancourtii* and *P. acanthamoebae* orthologous proteins and their 20 best blast hits, restricted to one representative per genus, where *Chlamydiales* or *Legionellales* representatives are directy related to other intracellular bacteria such as *Rickettsia, Ehrlichia, Orientia* or *Wolbachia*. In the last phylogenetic reconstruction *Legionella* clusters with another intra-amoebal bacterium, *Amoebophilus asiaticus.* Trees for ID 191, 192 and 387 are found in supplementary figure 1 and the tree for ID 263 is shown in supplementary figure 3. Sequences retrieved by using *L. drancourtii* or *P. acanthamoebae* protein as a query, are indicated with the prefix LLAP or PAH, respectively. Bacteria belonging to the *Legionellales, Chlamydiales* and *Rickettsiales* orders are shown respectively in blue, green and red.

**Supplementary Figure 5. Phylogenetic trees with eukaryotic representatives**

In two cases (A and C), eukaryotic sequences were identified by BLASTP homology searches. In another tree (B), *L. drancourtii* clustered with the amoeba-associated *Bacteroidetes* named *Amoebophilus asiaticus.* Maximum likelihood trees of *L. drancourtii* and *P. acanthamoebae* orthologous proteins were build using their 20 best blast hits, restricted to one representative per genus. Sequences retrieved by using *L. drancourtii* or *P. acanthamoebae* protein as a query, are indicated with the prefix LLAP or PAH, respectively. Bacteria belonging to the *Legionellales, Chlamydiales* and *Rickettsiales* orders are shown respectively in blue, green and red.

**Supplementary Figure 6. Genic GC content**

The genic GC contents of *P. acanthamoebae* (A) and *L. drancourtii* (B) are shown in grey, whereas orthologous genes are shown in light pink. Horizontally transferred genes are colored by categories of the putative partners according to the legend within the figure: in blue-green between *L. drancourtii* and *P. acanthamoebae*; in purple between *Legionellales* and *Chlamydiales* members; in pink between *L. drancourtii* or *P. acanthamoebae* and *Rickettsiales*; in yellow with Eukaryotes or *A. asiaticus.* Panels (C) and (D) present the genic GC content at the 3^rd^ position of the codon respectively in *P. acanthamoebae* and *L. drancourtii* using a similar color-code.

**Supplementary Figure 7. Percentage identity and coverage in *L. drancourtii* and *P. acanthamoebae* orthologs.**

Proteins potentially transferred horizontally are colored according to four categories of gene transfer: in blue-green between *L. drancourtii* and *P. acanthamoebae*; in purple between *Legionellales* and *Chlamydiales* members; in pink between *L. drancourtii* or *P. acanthamoebae* and *Rickettsiales*; in yellow with Eukaryotes or *A. asiaticus.*

SUPPLEMENTARY TABLE LEGENDS

**Supplementary Table 1. Conserved orthologous genes**

This table lists the 208 orthologs of *L. drancourtii* (LLAP) and *Pr. acanthamoebae* (PAH) also found in the genomes of *P. amoebophila*, *C. trachomatis*, *R. baltica*, *L. pneumophila*, *C. burnetii*, and *E. coli*.

**Supplementary Table 2. Orthologous genes identified in 5 bacteria**

This table presents the 80 orthologs of *L. drancourtii* (llap) and *P. acanthamoebae* (pah) identified in five among the six bacterial genomes screened (*P. amoebophila*, *C. trachomatis*, *R. baltica*, *L. pneumophila*, *C. burnetii*, and *E. coli)*. The last column indicates the bacterial genome where the corresponding gene could not be identified.

**Supplementary Table 3. Orthologous genes identified in four bacteria**

Accession number and annotation of the 77 orthologs of *L. drancourtii* (LLAP) and *P. acanthamoebae* (PAH) also identified in four of the six bacterial genomes screened (*Pr. amoebophila*, *C. trachomatis*, *R. baltica*, *L. pneumophila*, *C. burnetii*, and *E. coli)*.

**Supplementary Table 4. Orthologous genes identified in three bacteria**

Accession number and annotation of the 59 orthologs of *L. drancourtii* (LLAP) and *P. acanthamoebae* (PAH) also identified in three of the six bacterial genomes screened (*Pr. amoebophila*, *C. trachomatis*, *R. baltica*, *L. pneumophila*, *C. burnetii*, and *E. coli)*.

**Supplementary Table 5. Orthologous genes identified in two bacteria**

Accession number and annotation of the 37 orthologs of *L. drancourtii* (LLAP) and *P. acanthamoebae* (PAH) also detected in two among the six bacterial genomes screened (*Pr. amoebophila*, *C. trachomatis*, *R. baltica*, *L. pneumophila*, *C. burnetii*, and *E. coli)*. The last column indicates the two bacterial species that harbour the corresponding homologous gene.

**Supplementary Table 6. Orthologous genes identified in one single bacterium**

Accession number and annotation of the 27 orthologs of *L. drancourtii* (llap) and *P. acanthamoebae* (pah) identified in only one of the six bacterial genomes screened (*Pr. amoebophila*, *C. trachomatis*, *R. baltica*, *L. pneumophila*, *C. burnetii*, and *E. coli)*. The last column indicates the bacterial species that harbours the corresponding homologous gene.

**Supplementary Table 7. Orthologous genes not identified in any bacteria screened**

Accession number and annotation of the 20 orthologs of *L. drancourtii* (LLAP) and *P. acanthamoebae* (PAH) that could not be detected in any of the six bacterial genomes screened (*Pr. amoebophila*, *C. trachomatis*, *R. baltica*, *L. pneumophila*, *C. burnetii*, and *E. coli)*.
