# Supplementary figures and images for: Insight into cross-talk between intra-amoebal pathogens
Source: BMC Genomics. 2011 Nov 2;12:542. doi: 10.1186/1471-2164-12-542 (PMC3220658; doi:10.1186/1471-2164-12-542)

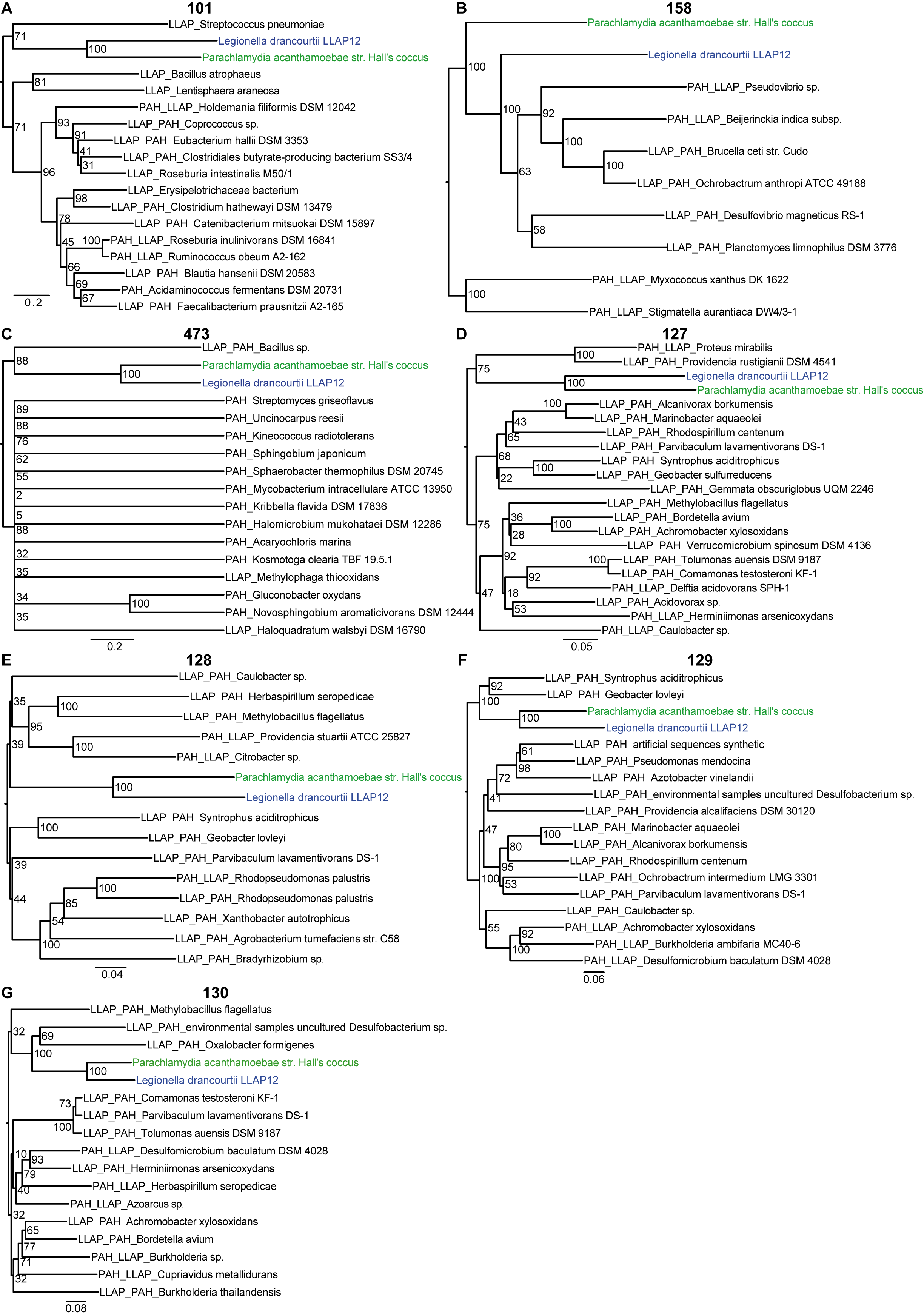

Supplement: Additional file 9 — Figure S1. Phylogenetic trees clustering L. drancourtii and P. acanthamoebae. Maximum likelihood trees of L. drancourtii and P. acanthamoebae orthologous proteins and their 20 best blast hits, restricted to one representative per genus. In these phylogenetic reconstruction, L. drancourtii and P. acanthamoebae cluster together. Sequences retrieved by using L. drancourtii or P. acanthamoebae protein as a query, are indicated with the prefix LLAP or PAH, respectively. Bacteria belonging to the Legionellales, Chlamydiales and Rickettsiales are shown respectively in blue, green and red. [file 1471-2164-12-542-S9.TIFF]

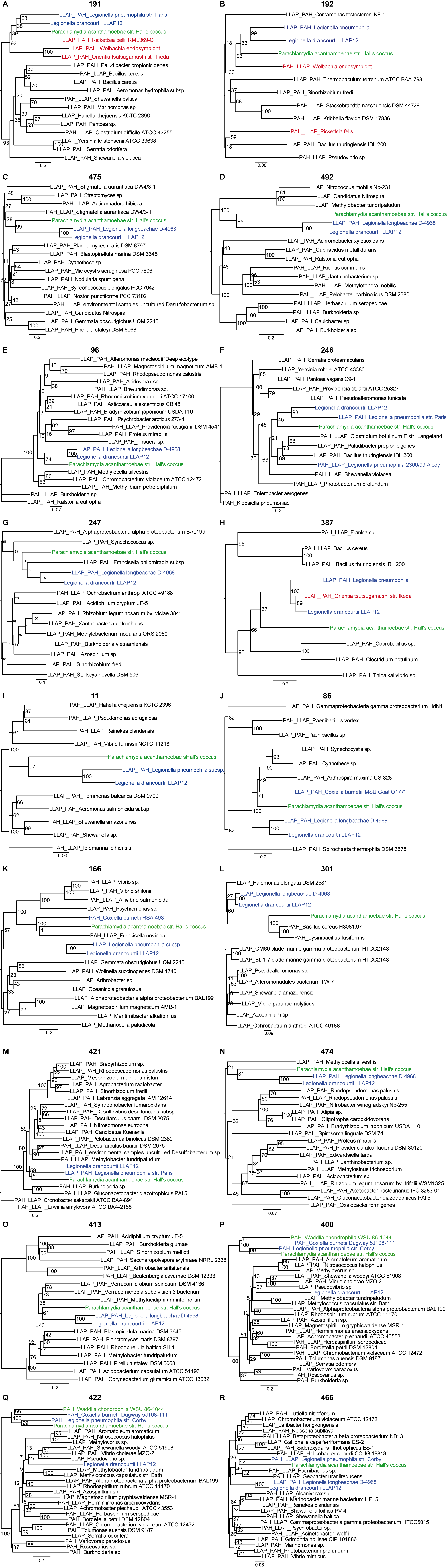

Supplement: Additional file 10 — Figure S2. Phylogenetic trees clustering P. acanthamoebae and Legionellales. Maximum likelihood trees of L. drancourtii and P. acanthamoebae orthologous proteins and their 20 best blast hits, restricted to one representative per genus. (A-O) P. acanthamoebae directly branches with Legionellales and (O-R) P. acanthamoebae clusters with L. pneumophila but more distantly to other Legionellales. Sequences retrieved by using L. drancourtii or P. acanthamoebae protein as a query, are indicated with the prefix LLAP or PAH, respectively. Bacteria belonging to the Legionellales, Chlamydiales and Rickettsiales are shown respectively in blue, green and red. [file 1471-2164-12-542-S10.TIFF]

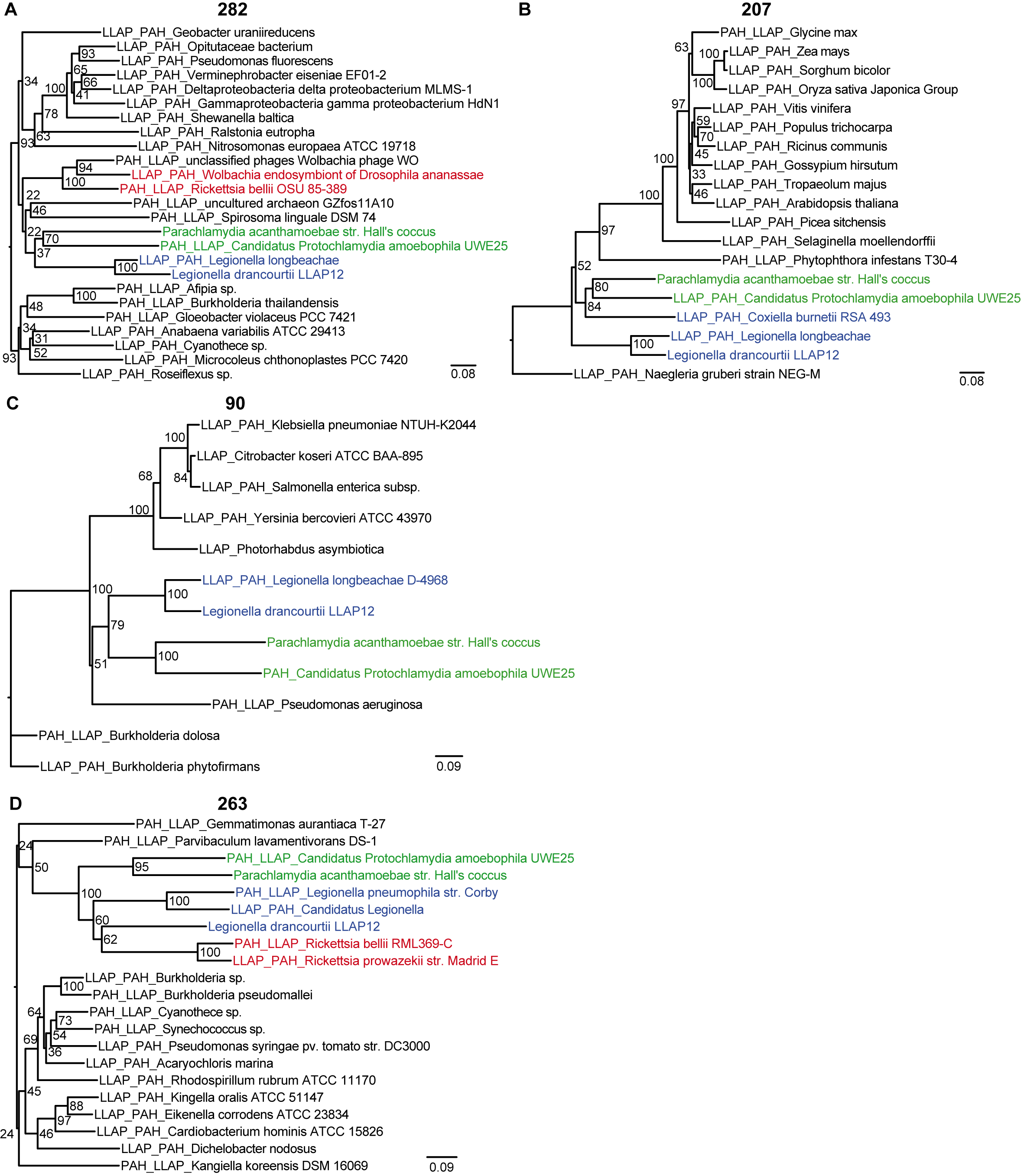

Supplement: Additional file 11 — Figure S3. Phylogenetic trees clustering Chlamydiales and Legionellales. Maximum likelihood trees of L. drancourtii and P. acanthamoebae orthologous proteins and their 20 best blast hits, restricted to one representative per genus, where bacteria of the Chlamydiales order and the Legionellales are directy related. Sequences retrieved by using L. drancourtii or P.acanthamoebae protein as a query, are indicated with the prefix LLAP or PAH, respectively. Bacteria belonging to the Legionellales, Chlamydiales and Rickettsiales orders are shown respectively in blue, green and red. [file 1471-2164-12-542-S11.TIFF]

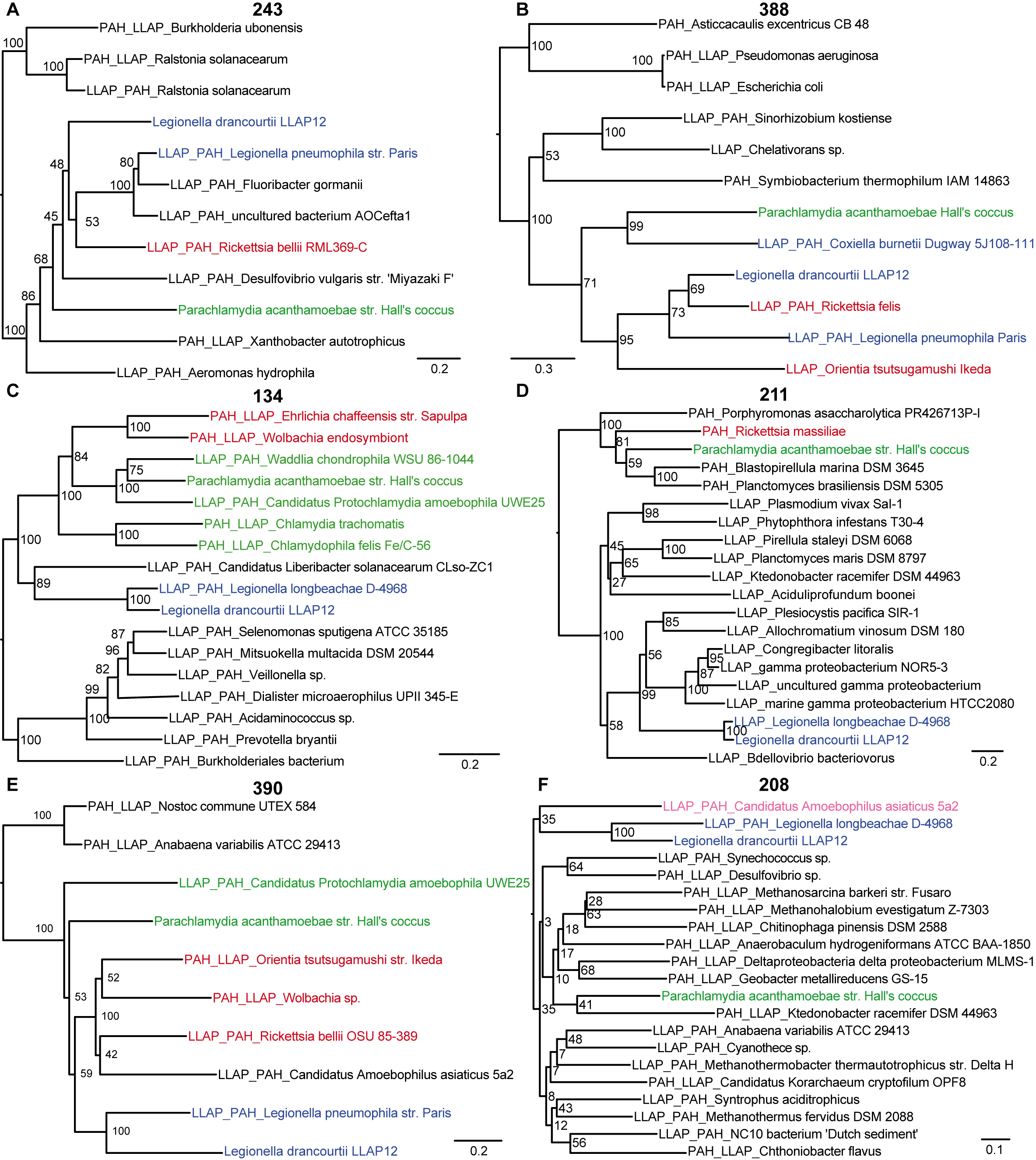

Supplement: Additional file 12 — Figure S4. Phylogenetic trees clustering L. drancourtii or P. acanthamoebae with Rickettsiales and A. asiaticus. Maximum likelihood trees of L. drancourtii and P. acanthamoebae orthologous proteins and their 20 best blast hits, restricted to one representative per genus, where Chlamydiales or Legionellales representatives are directy related to other intracellular bacteria such as Rickettsia, Ehrlichia, Orientia or Wolbachia. In the last phylogenetic reconstruction Legionella clusters with another intra-amoebal bacterium, Amoebophilus asiaticus. Trees for ID 191, 192 and 387 are found in Additional file 2, Figure S1 and the tree for ID 263 is shown in Additional file 4, Figure S3. Sequences retrieved by using L. drancourtii or P. acanthamoebae protein as a query, are indicated with the prefix LLAP or PAH, respectively. Bacteria belonging to the Legionellales, Chlamydiales and Rickettsiales orders are shown respectively in blue, green and red. [file 1471-2164-12-542-S12.TIFF]

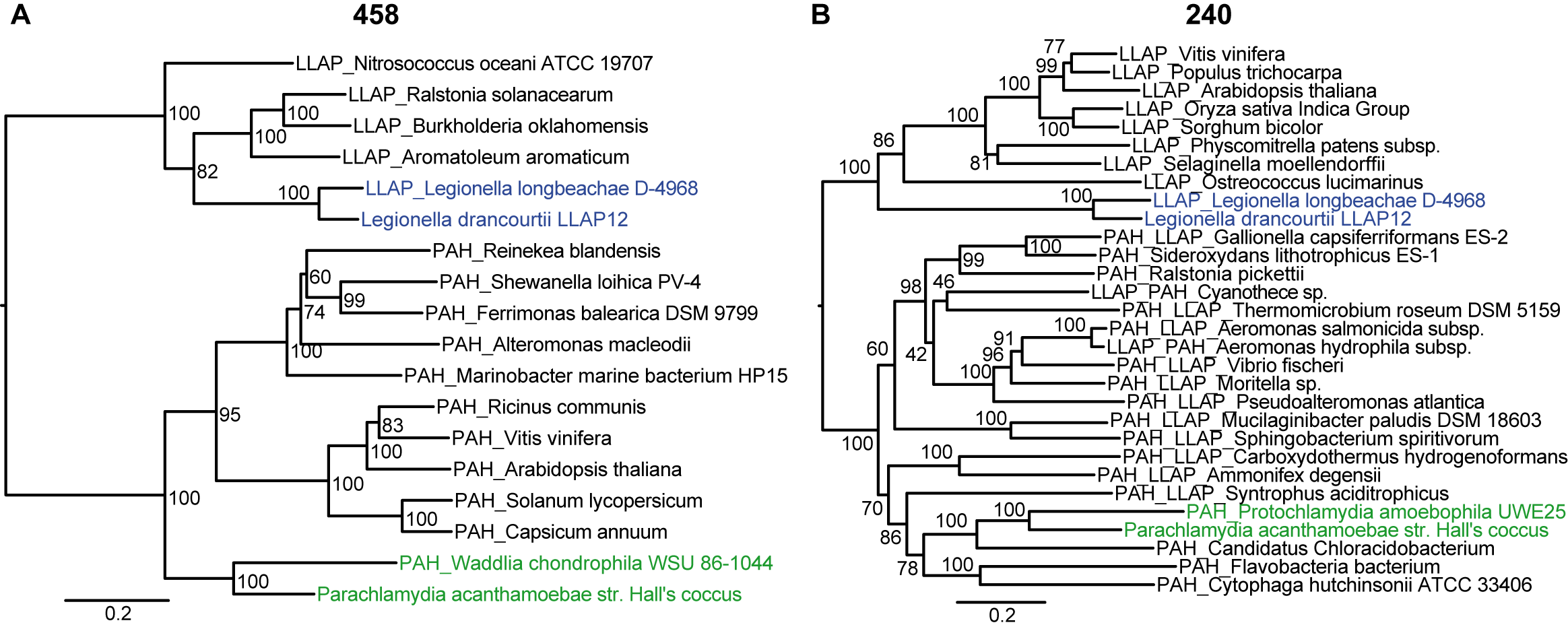

Supplement: Additional file 13 — Figure S5. Phylogenetic trees with eukaryotic representatives. In two cases (A and C), eukaryotic sequences were identified by BLASTP homology searches. In another tree (B), L. drancourtii clustered with the amoeba-associated Bacteroidetes named Amoebophilus asiaticus. Maximum likelihood trees of L. drancourtii and P. acanthamoebae orthologous proteins were build using their 20 best blast hits, restricted to one representative per genus. Sequences retrieved by using L. drancourtii or P. acanthamoebae protein as a query, are indicated with the prefix LLAP or PAH, respectively. Bacteria belonging to the Legionellales, Chlamydiales and Rickettsiales orders are shown respectively in blue, green and red. [file 1471-2164-12-542-S13.TIFF]

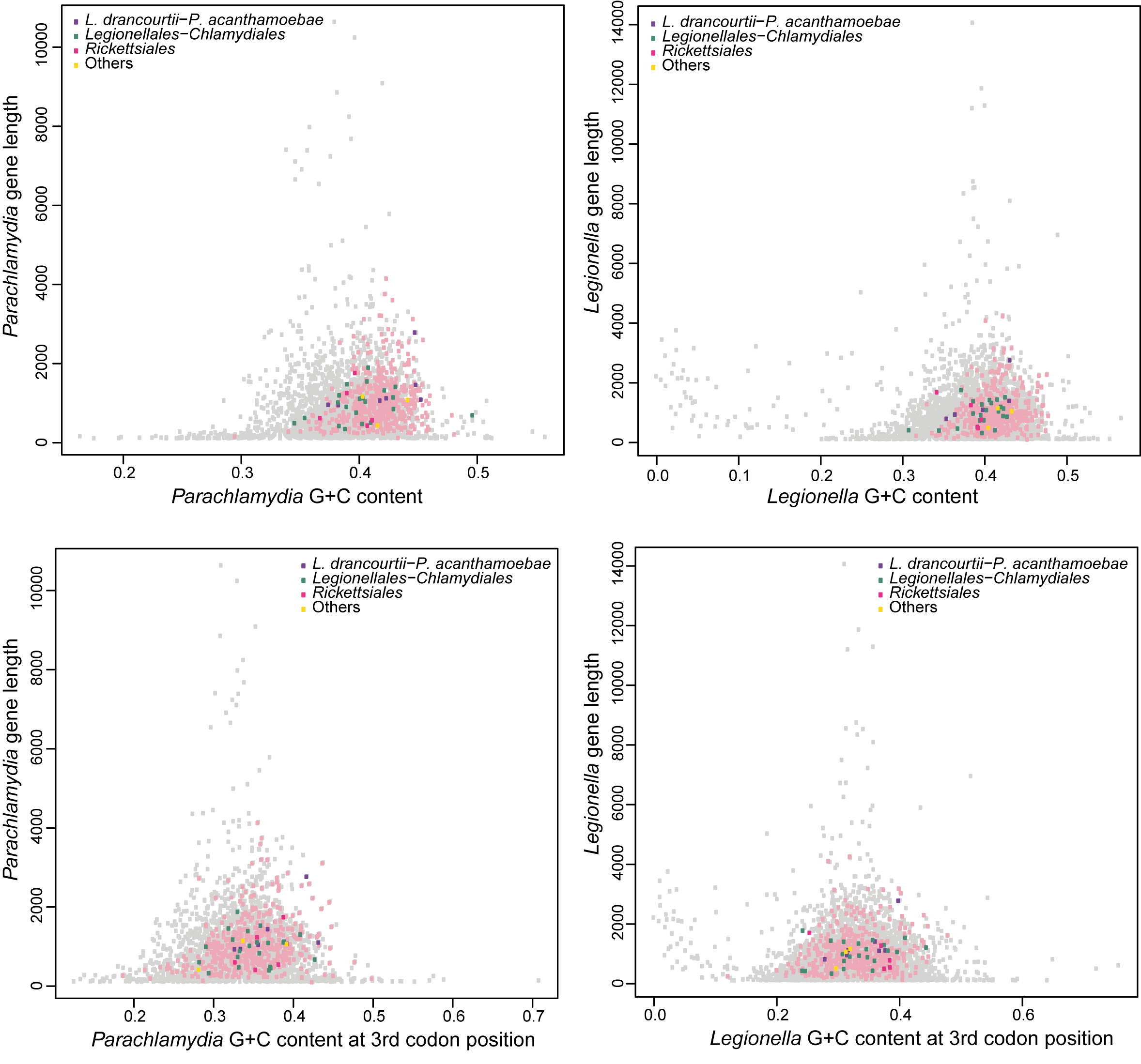

Supplement: Additional file 14 — Figure S6. Genic GC content. The genic GC contents of P. acanthamoebae (A) and L. drancourtii (B) are shown in grey, whereas orthologous genes are shown in light pink. Horizontally transferred genes are colored by categories of the putative partners according to the legend within the figure: in blue-green between L. drancourtii and P. acanthamoebae; in purple between Legionellales and Chlamydiales members; in pink between L. drancourtii or P. acanthamoebae and Rickettsiales; in yellow with Eukaryotes or A. asiaticus. Panels (C) and (D) present the genic GC content at the 3rd position of the codon respectively in P. acanthamoebae and L. drancourtii using a similar color-code. [file 1471-2164-12-542-S14.TIFF]

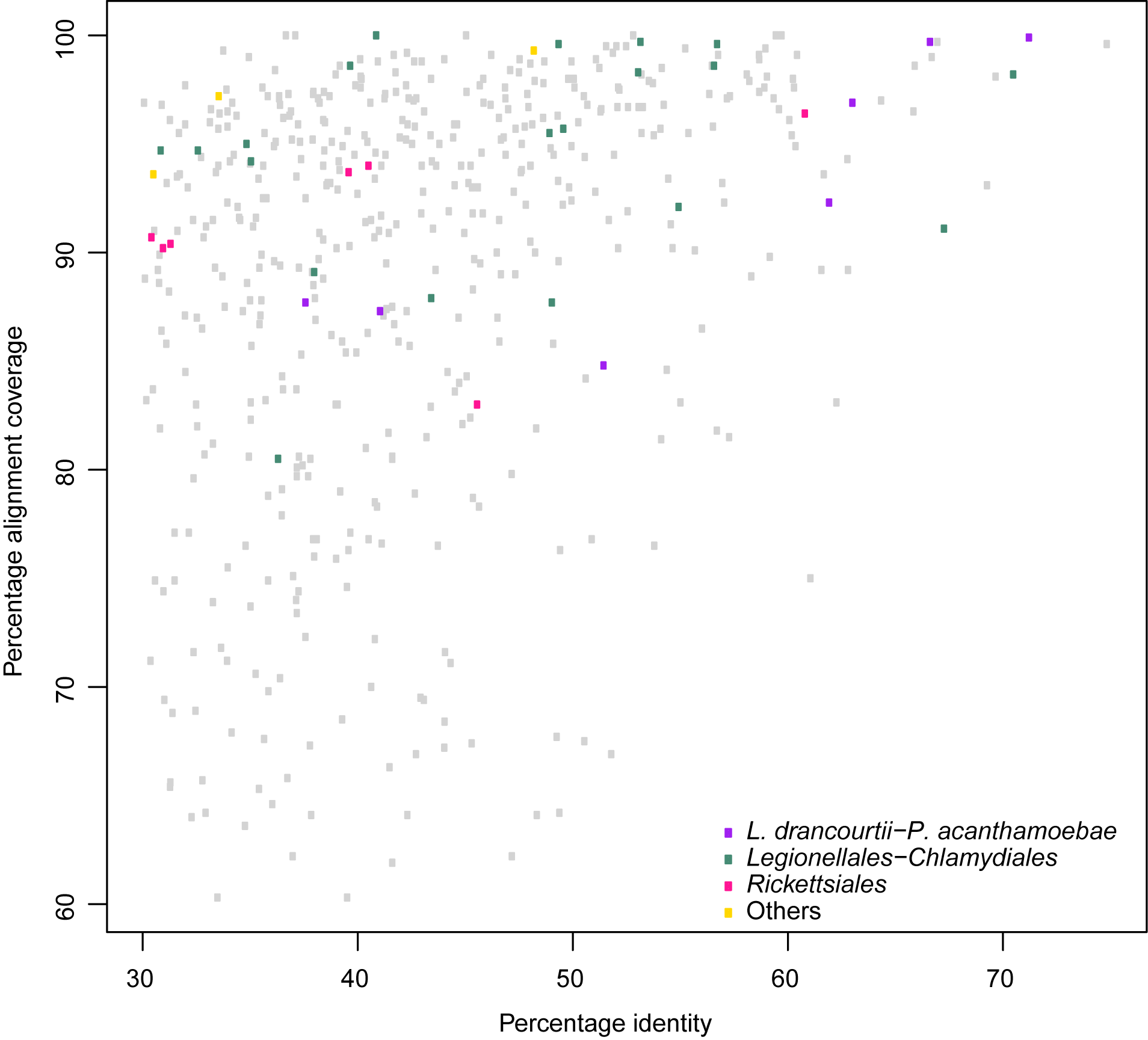

Supplement: Additional file 15 — Figure S7. Percentage identity and coverage in L. drancourtii and P. acanthamoebae orthologues. Proteins potentially transferred horizontally are colored according to four categories of gene transfer: in blue-green between L. drancourtii and P. acanthamoebae; in purple between Legionellales and Chlamydiales members; in pink between L. drancourtii or P. acanthamoebae and Rickettsiales; in yellow with Eukaryotes or A. asiaticus. [file 1471-2164-12-542-S15.TIFF]
